# Understanding the contemporary high obesity rate from an evolutionary genetic perspective

**DOI:** 10.1186/s41065-023-00268-x

**Published:** 2023-02-07

**Authors:** Tong Wu, Shuhua Xu

**Affiliations:** 1 State Key Laboratory of Genetic Engineering, Center for Evolutionary Biology, Collaborative Innovation Center of Genetics and Development, School of Life Sciences, Fudan University, Shanghai, 200438 China; 2 Human Phenome Institute, Zhangjiang Fudan International Innovation Center, and Ministry of Education Key Laboratory of Contemporary Anthropology, Fudan University, Shanghai, 201203 China; 3 Department of Liver Surgery and Transplantation, Liver Cancer Institute, Zhongshan Hospital, Fudan University, Shanghai, 200032 China

**Keywords:** Obesity, Evolutionary genetics, Thrifty gene hypothesis, Drifty gene hypothesis, Maladaptation viewpoint, Genome-wide association study

## Abstract

The topic of obesity is gaining increasing popularity globally. From an evolutionary genetic perspective, it is believed that the main cause of the high obesity rate is the mismatch between environment and genes after people have shifted toward a modern high-calorie diet. However, it has been debated for over 60 years about how obesity-related genes become prevalent all over the world. Here, we review the three most influential hypotheses or viewpoints, i.e., the thrifty gene hypothesis, the drifty gene hypothesis, and the maladaptation viewpoint. In particular, genome-wide association studies in the recent 10 years have provided rich findings and evidence to be considered for a better understanding of the evolutionary genetic mechanisms of obesity. We anticipate this brief review to direct further studies and inspire the future application of precision medicine in obesity treatment.

## Background

It has been reported that 10.8% of males and 14.9% of females were obese (with body mass index [BMI] > 30) in 2014, which was based on over 19 million people from more than 200 different countries and districts. There are over six trillion people with obesity, and the number is still increasing [1]. Obesity is related to a variety of noncommunicable chronic diseases (NCD), such as hypertension, cardiovascular disease, type 2 diabetes (T2D), and certain kinds of cancer [2–4]. In 2019, obesity-related disease contributed to 11.1% of the death toll caused by NCD [5]. Obesity treatment has become one of the main medical costs in many countries; for instance, in the United States, costs reached 147 trillion dollars in 2008 [6]. However, there is so far still no efficient medicine to treat obesity [7]. Therefore, it is necessary to unravel the mechanism of obesity and promote the development of obesity treatment.

The cause of obesity is very complex and includes maternal physiological status (maternal nutrition can change fetal epigenetic programming and influence the susceptibility of fetuses to obesity [8, 9]), self-physiological status (such as dietary structure, feeding pattern, exercise, and hormone level), and genetic factors (many gene loci have been found to affect BMI [10–14]). Genetic factors play a key role [15], given that a study involving over 25,000 pairs of twins and 50,000 adoptive families has shown that the influence of genetic factors on obesity was 50%–90% and 20%–80%, respectively [16].

The obesity epidemic has only occurred in the recent 100 years of human evolution history [17]. About 2.5 million years ago (mya), hominids developed into the paleolithic age, living on a hunter-gatherer pattern. Around 10,000 years ago, humans shifted from the paleolithic age to the Neolithic age and started an agricultural society, in which people mainly fed on crops. It was in the 1860s that the industrial revolution started and brought enormous change to humans. Especially, the modern high-calorie diet has gradually gained popularity [18].

Because of the great influence of genetic factors on obesity, researchers have been trying to explain the epidemic from an evolutionary perspective. There is an agreement that the obesity epidemic is caused by a mismatch between environment and genes, as the human genome unlikely changes rapidly in 100 years. However, there is an ongoing debate when it comes to how obesity-related genes are prevalent. There are mainly three hypotheses, including the thrifty gene hypothesis, the drifty gene hypothesis, and the maladaptation viewpoint. As the latest review on this topic [19] and all related reviews are written in adherence to certain hypotheses [17, 19–23], this paper overviews all influential papers about the abovementioned three hypotheses and combines the latest genome-wide association studies (GWAS) as factual evidence for corresponding hypotheses [24–28], to hopefully facilitate understanding of the obesity epidemic from an evolutionary perspective and provide direction for future studies towards precision medicine.

## The thrifty gene hypothesis

In 1962, Neel first proposed the “thrifty gene hypothesis” to explain the high rate of obesity and related diseases from an evolutionary perspective. The hypothesis was initially raised to understand diabetes and was extended later. According to Neel, before industrial society, people frequently suffered famine for the lack of secured food resources. People with the “thrifty gene” could use food energy more efficiently and therefore avoid the problems caused by famine, such as death and low fecundity. In this way, famine posed a positive selection on the thrifty gene, and thus increased the gene frequency. However, after the industrial revolution, food was guaranteed and dietary structure shifted toward the high-calorie one. Therefore, the thrifty gene’s attribute of efficiently using food energy has led to obesity and related diseases [29, 30]. Arguments and corresponding criticism are mainly from factual and logical levels.

### Does the thrifty gene exist?

It would be strong proof of the thrifty gene hypothesis if certain variants, such as single-nucleotide polymorphism (SNP) associated with obesity as identified by GWAS showed evidence for positive selection and were proven on biochemical and physiological levels to regulate gene expression to better use food energy. In 2020, a study published in *Cell* successfully provided such proof for the first time. It was found that the *2q21.3* locus on human chromosome 2 has an extremely long haplotype (> 1 Mb), high gene frequency (about 77%), and high F_ST_ value (indicating highly different gene frequency among different populations) by comparing 101 SNPs, which is a typical signature of positive selection [31]. A phenome-wide association study showed that the European *2q21.3* variant was significantly associated with several obesity-related attributes, such as whole-body fat mass, trunk fat mass, and leg fat mass. Moreover, rs1438307 is located at a *miR-128-1* promoter. Hi-C indicates that the European variant can increase the accessibility of *miR-128-1* towards DNase I, promoting the expression of *R3HDM1*, as *miR-128-1* is an intronic miRNA in the *R3HDM1* gene. The *R3HDM1* gene is related to both increased feeding efficiency and increased intramuscular fat. It has been proven in animal experiments that miR-128-1 can improve metabolic status and relieve insulin resistance and inflammation in obese mice by controlling metabolic gene expression, glucose homeostasis, and energy expenditure in metabolic tissues [27].

However, *miR-128-1* is the only case so far as solid evidence supporting the existence of the thrifty gene. Most studies have only provided indirect evidence. For example, the *PPARA* gene encodes a nuclear receptor, which regulates a variety of genes associated with glucose homeostasis and lipid metabolism [32] and is activated when lacking energy [33]. At the same time, L162V and rs4253728 at the *PPARA* gene show significant differences among populations and are associated with high lipid metabolism as revealed by GWAS analysis [34]. Moreover, an analysis of 120 Wolaita genomes with integrated haplotype score (iHS) and cross-population extended haplotype homozygosity (XP-EHH) showed several selection signals. It is worth noting that 10,000 years ago, because of a food crisis, the ancestors of the population for a long period fed on *Ensete ventricosum*, which contains high glucose and low lipid, corresponding to PPARA’s function [35]. The evidence seemingly implies that *PPARA* is a thrifty gene, but the abovementioned two studies investigated different SNPs; therefore, they cannot provide conclusive support for the thrifty gene hypothesis. Likewise, the *IRS2* gene plays a key role in pancreatic beta-cell development and IGF1 receptor-mediated insulin signaling. *IRS2*^–/–^ mice suffer from serious T2D caused by insulin resistance [36]. A common variant in human G1057D is also significantly associated with T2D [37]. At the same time, a study of the 1000 Genomes data and the HapMap data has demonstrated that among African and European populations, the *IRS2* locus is under evident positive selection (|iHS|> 2.5) [24]. Nevertheless, the abovementioned SNPs are also not identical.

The lack of factual evidence becomes the main target of criticism. In 2009, Southam first found 30 obesity and related disease-associated SNPs with a genome-wide statistical significance threshold of *p* < 5 × 10^–8^, and then oppugned in three ways. First, according to the thrifty gene hypothesis, thrifty genes appear after agricultural society (explained in detail later), but the alignment of human and chimpanzee gene sequences showed that most SNPs (13 in 30) exist in chimpanzees. Second, the hypothesis indicates that different environments pose different selection pressures, so SNPs are expected to be different in frequency among different populations. However, among 30 SNPs, only one SNP at *TCF7L2* meets the anticipation with an F_ST_ value of 0.579. Third, only two in 30 SNPs reach the threshold of |iHS|> 2. In other words, most obesity-related SNPs found in this study show no signals of natural selection [38]. Similarly, in 2016, Wang and Speakman estimated seven different statistics, including Tajima’s D, linkage disequilibrium, high-frequency derived allele, F_ST_, cross-population composite likelihood ratio, iHS, and XP-EHH in fourteen populations in the 1000 Genomes Project, and found that only nine in 115 BMI-associated SNPs are under positive selection, among which five SNPs are associated with decreasing BMI [20].

Notably, although the criticism that thrifty genes are too few to support the hypothesis does make sense, it is not a fundamental challenge. Logically, the existence of a thrifty gene is just a necessary condition for the thrifty gene hypothesis. In other words, to prove the hypothesis, we just need to argue that there is at least even only one single thrifty gene, rather than that all or most obesity-related genes are thrifty genes, because the correctness of the thrifty gene hypothesis does not exclude other factors, as other factors can also increase obesity-related gene frequency. Therefore, the amount of the thrifty genes only affects the importance of the thrifty gene hypothesis, but not its correctness.

### Can famine pose selection pressure?

Speakman gave a strong logical criticism of the thrifty gene hypothesis in 2008. His statement began with the duration of selection pressure posed by famine [21]. There are mainly two opinions among champions of the hypothesis. Some support that famine selection pressure on humans exists from 2 mya, which was proposed by Neel, the founder of the thrifty gene hypothesis. Chakravarthy later provided some supporting materials [39] that there was no significant change in human mitochondrial DNA [40]. Nevertheless, Neel discovered that the Pima are not susceptive to diabetes, and thus changed his idea 27 years after the initial proposal, and believed that positive selection occurred after agricultural society [41]. The most convincing argument for it was put forward by Prentice. He stated that 2 mya, humans were still in the Neolithic age, living on the hunter-gatherer pattern. Although there was a frequent lack of food, as food sources were diverse and people were not settled, lethal famine rarely happened. However, about 10,000 years ago, after humans developed agriculture, they relied heavily on crops. As long as the climate was unsuitable for crops, catastrophic famine, such as the Great Famine, could happen, causing large-scale death and posing sufficient selective pressure [30].

Based on the abovementioned viewpoints, Speakman rebutted Haldane’s formula in two ways. The first starts from the assumption that the thrifty gene is a dominant variant (A) over the other allele (a) with a selective advantage (*k*) of 0.001, i.e., an individual with the A allele has 0.1% survival or fecundity advantage over aa, then the frequency of thrifty genes are expected to increase from 1 to 99% in 16,500 generations. However, if selective pressure exists from the beginning of human history, which is 2 million years, or 100,000–70,000 generations, then all modern humans should have thrifty genes, but the fact is that the obesity rate is 20%–30%. If selective pressure started from an agricultural society, then it only went through 400–600 generations, too short for a thrifty gene to be effectively dispersed among modern humans.

The other demonstration starts from the prediction of the thrifty gene hypothesis that after 600 generations, the gene frequency is expected to rise from 1 to 30%. On the one hand, to meet the abovementioned prediction, the *k* value should be 0.03, but Speakman argued that positive selection posed by famine could not reach the value. Although food shortages happened once every 10 years, most of them did not cause death. Catastrophic famines generally happened once every 5–7 generations. Therefore, the *k* value should be 0.03 × 5 to 0.03 × 7, which is 15%–21%. On the other hand, data showed that the death rate of great famines was 5%–12%, which could not reach the required *k* value. Furthermore, usually, it was the older adults and children who died in a famine. In most cases, the older adults had already had their descendants, while children were rarely fat in any population (childhood obesity did not exist until recent 50 years). Therefore, even vulnerable people of famine did not contribute to differentiated death rates among different BMI groups [42].

One year later, Prentice responded directly by oppugning the way that famines posed selective pressure as Speakman claimed. Speakman’s statement is based on the assumption that selective pressure came only from death caused by famines. However, Prentice believed that lowered fecundity in famines was also an influential factor. The decrease in body fat would inhibit leptin secretion, which would then lower fecundity through the hypothalamic-pituitary–gonadal axis [43]. Data are also supportive, as famines that happened in Cumberland and Westmorland in 1597 and 1623 led to a 47.6% and 68.6% decrease in fertility rate, respectively [44]. Moreover, Fisher's fundamental theorem of natural selection, a well-accepted theory, also directly points out that the change in fertility rate can form an effective selective pressure within a short period [45]. Therefore, not only is Speakman’s calculation invalid, but his other assumptions might also be wrong. First, rather than once every 5–7 generations, influential famine happened annually. For example, in the Gambia, the birth rate reached the lowest point routinely in the ninth month after a food shortage every year [44]. Second, vulnerable people in famines include not only older people and children but also people of reproductive age, as supported by the Gambia data [23].

Speakman responded and pointed out that although the birth rate decreased during famines, it bounced back even higher than normal, thereby offsetting the influence of famine. In addition, several studies have shown that famines impaired fecundity, not through fat insufficiency, but through sudden energy imbalance and intracellular oxidation state change, so famines are expected to impact differently among different BMI groups [21]. Speakman rebutted further that if the thrifty gene hypothesis was valid, then between two famines, BMI should be higher, but neither hunter-gatherer society nor agricultural society showed such a pattern [42]. Prentice later proposed that in the Gambia, female BMI does rise between two famines, contradicting Speakman directly [46].

## Drifty gene hypothesis

A half-century after Neel proposed the thrifty gene hypothesis, Speakman raised an explanation for the present high obesity rate, i.e., the drifty gene hypothesis. He argued that since humans mastered weapons and fire, they eliminated the selective pressure of predators, and thus arbitrary gene mutation and drift that cause obesity can be passed down from generation to generation [21].

### Dual-intervention point model

The drifty gene hypothesis was initially established on the dual-intervention point model proposed by Speakman in 2007. According to the model, there are mainly two factors of body mass, the upper intervention point (being hunted by predators) and the lower intervention point (being too lean to survive food shortage) [47]. In 2008, Speakman claimed that the lower intervention point could not contribute enough selective pressure (refer to the abovementioned criticism for the thrifty gene hypothesis). Instead, the true reason for today’s obesity epidemic is the drift and mutation of the upper intervention point-related genes. First, 2–4 mya, hominids (such as *Australopithecus*), who weighed only 29–52 kg and lived in Sahara, were seriously threatened by large-size predators. For example, *Dinofelis* was demonstrated by isotope detection to feed on *Australopithecus*, forming unneglectable selective pressure. However, 2 mya, *Homo erectus*, who had a larger society and body size, evolved and acquired tools and fire for protection. Meanwhile, 1.4 mya, *Dinofelis,* as well as most other large carnivores, are distinct in East Africa. Therefore, Speakman believes that predators had a decreasing effect on overweight (i.e., the upper intervention point was disappearing), and obesity-related genes produced by gene mutation and drift were kept throughout the remaining evolution history of humans [21].

**Fig. 1:**
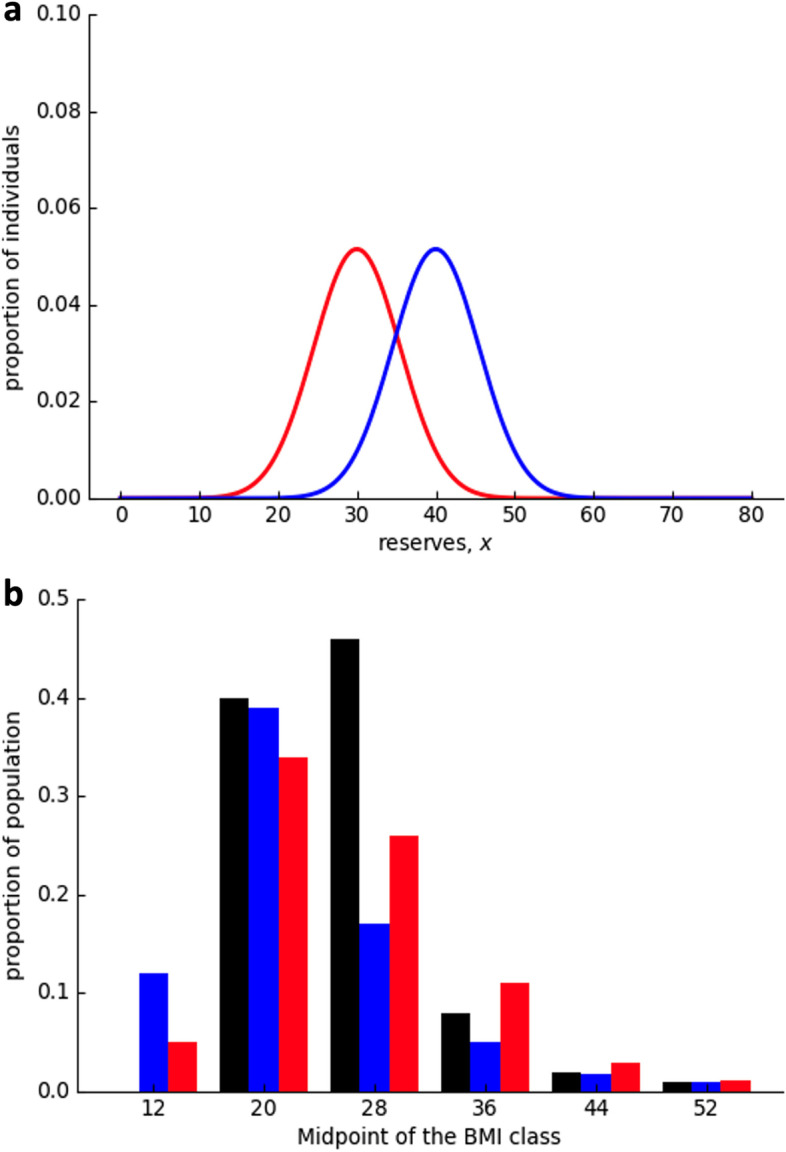
The dual-intervention point model predicts BMI distribution (modified from Higginson (2016 [48])). **a** Average BMI (reserves) changes with environment, and variance keeps still according to Higginson; **b** Factual BMI distribution pattern (black), the dual-intervention point model prediction (blue), and the Higginson modified-dual-intervention point model prediction (red)

Speakman provided quantitative analysis and found that the prediction generally complied with the present BMI distribution. There were four assumptions of the calculation model: (i) the upper intervention point is influenced by multiple independent genes, which can be simplified to one major gene; (ii) referring to leptin’s impact on obesity, assuming a major gene allele can affect 8 BMI units; (iii) referring to the existing paleontology studies, taking the average BMI of human 1.8 mya as 20; (iv) arbitrary gene mutation takes place once every generation. According to the drifty gene hypothesis, while the upper intervention point has been invalid, the lower intervention point is still functioning (note: Speakman’s viewpoint when rebutting the thrifty gene hypothesis that famine was not powerful enough to pose selective pressure is not paradoxical, because Speakman did not deny that underweight caused by famine was fatal, but that the possibility of death in famine was high enough); therefore, underweight people are eliminated, while overweight ones survive. Overall, combined with the Poisson distribution, the theoretical BMI distribution generally complied with the fact (Fig. 1c). This hypothesis is also the first to explain a small group of extreme obesity (BMI > 40). Nevertheless, Speakman himself admitted that defects still exist. The proportion of the extremely obese group was relatively too high, probably because of the incorrect assumption that a single “major obesity-related gene” affects 8 BMI units. If substituting that with 20 minor genes affecting 0.1 BMI units, the extremely obese group drops dramatically; if 160 minor genes affect 0.1 BMI units, then there will be far more extremely obese people [47].

Furthermore, the dual-intervention point model—the basis of the thrifty gene hypothesis—was challenged by Higginson in two aspects. The first challenge is that the predator's threat never disappears. Although the usage of weapons and fire did lower the risk of being killed, there is no evidence to support the idea that the risk was eliminated. Additionally, being overweight decreases sexual attractiveness, so the upper intervention point still poses pressure. Moreover, according to the dual-intervention point model, body mass is unchanged within the range, but behavioral ecology study reveals that the potential risk of hunger and hunting will also affect body weight even within the range of the dual-intervention point, and thus body mass still floats. For instance, if the cost of food increases, to improve the competence in hunting, one’s weight will decrease gradually to a lower intervention point. The phenomenon that BMI does not change within the dual-intervention point range is not because every individual’s weight keeps still. Rather, it is because every individual’s weight changes differently and statistically appear to be stationary in general. The second challenge is that the two intervention points are not independent. When either predation or hunger risk changes, both intervention points will change accordingly, that is, when predation risk increases, both upper and lower intervention points increase accordingly. Therefore, the BMI distribution pattern’s location changes with the environment, but not its shape (Fig. 1a). If calculated with a modified dual-intervention point model, then the prediction will be different from the fact (Fig. 1b) [48].

### Disease-predation dual-intervention point model

Facing Higginson’s criticism, Speakman responded in 2018 and revised the model to the disease-predation dual-intervention point model. For the upper intervention point, Speakman took Higginson’s criticism and considered the selective pressure of being hunted. As for the viewpoint that body mass will be affected by the potential risk of predation and hunger, Speakman refuted that it implicated that all excess energy is stored in the form of fat, but the fact is that it is also stored in undigested food in the stomach and glycogen in muscle and liver, which is capable of dealing with short-term potential environment change without changing body mass [19]. Inspired by it, Speakman considered glycogen and fat’s influence on hormones, such as ghrelin, PYY, CCK, GIP, and leptin, and therefore regulation of food intake.

The theory about the lower intervention point was also modified. Instead of starving to death, Speakman believed that it was diseases that took effect. Although the insurance hypothesis raised in 2017 proposed that insecurity of food was a key factor in obesity based on 125 epidemiology studies, it was also challenged to be counterfactual. A study integrating 178 studies showed that with secured food resources, the animals generally gain fat [49], contradicting the insurance hypothesis. Speakman thus proposed a new explanation. Diseases weaken hunting ability [50] and promote the secretion of several cytokines to inhibit food intake [51–53] (note: the evidence provided by Speakman only suggested that certain cytokines have the function, but cytokines function differently in different situations, so cytokines do not necessarily inhibit food intake during sickness); therefore, food intake reduces, and those without enough fat storage are more vulnerable to death. Data are also supportive, as high body mass individuals are more likely to survive pneumonia [54]. Overall, considering disease and predation, as well as the inaccuracy of natural selection and self-regulation, Speakman raised the new disease-predation dual-intervention point model [19].

**Fig. 2:**
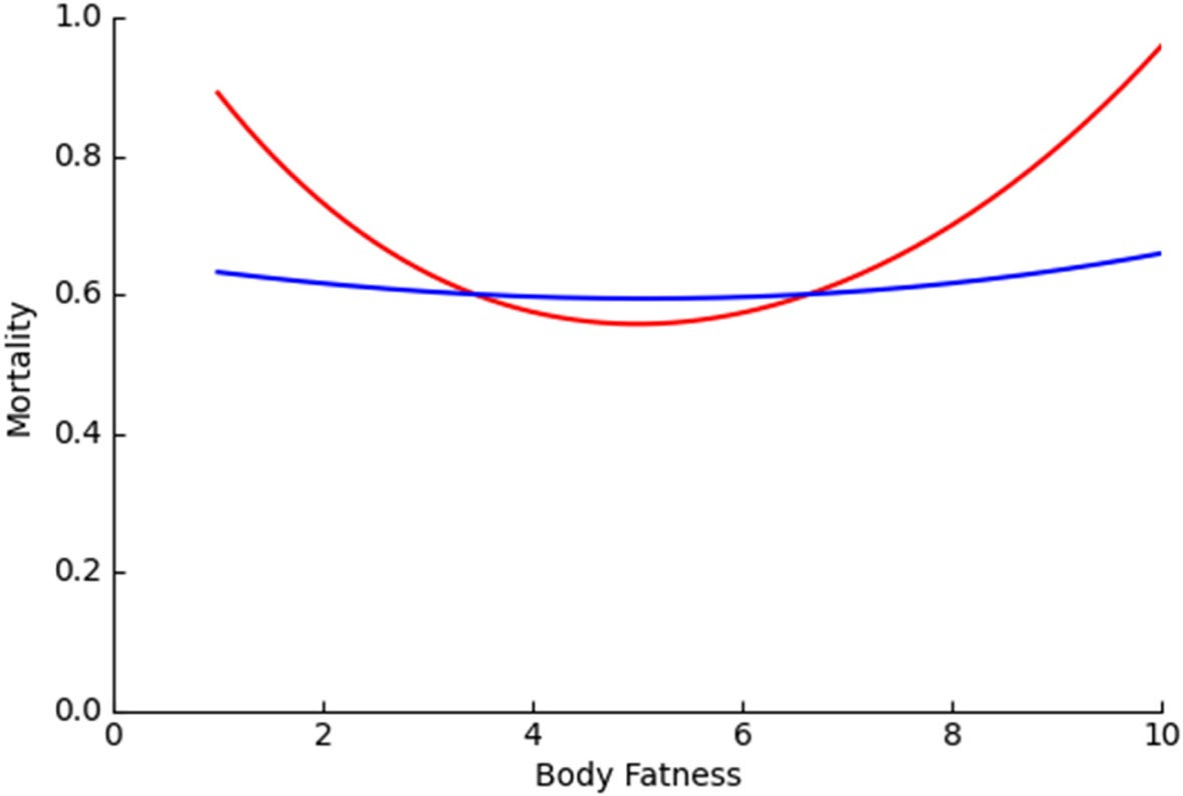
Prediction of the relationship between body fatness and mortality with the different parameter values (modified from Speakman (2018) [19]). The formula of the disease-predation dual-intervention point model is $a \times {e}^{-bF}+h+c \times {e}^{gF}+j$ (F stands for body fatness; a, b, c, e, h, and j are parameters). For red line, a = 1.0, b = 0.3, c = 0.123, g = 0.2, j = h = 0; For blue line, a = 0.53, b = 0.07, c = 0.123, g = 0.117, h = j = 0. Units for mortality and fatness are arbitrary

We believe that the model is still defective. First, there are three undefinable parameters. Different values result in different predictions (Fig. 2) and therefore greatly undermine the validity as it cannot be tested with reality. Second, the model assumes that body fatness is negatively related to disease lethality, but there are a variety of obesity-related diseases, such as cardiovascular disease and diabetes [55], which further lowers the credibility of the model.

## Maladaptation viewpoint

In 2014, Sellayah raised a brand-new viewpoint for today’s high obesity rate—the maladaptation viewpoint. While the thrifty gene hypothesis and the drifty gene hypothesis suggest that obesity-related gene is selected directly by famine, Sellayah believes that it is a by-product of the natural selection of climate. This is also the only viewpoint that provides a plausible explanation of regional differentiation of obesity incidence.

SNP sites that meet the following conditions can serve as solid evidence for the maladaptation viewpoint: (i) the SNP sites show signals of natural selection; (ii) they are associated with both climate and body fatness; (iii) the SNPs' genes affect climate adaptation and fat storage.

Eliminating the impact on the environment, economy, and education, the incidence of obesity and related diseases incidence still highly diverse among different populations [56–58]. Moreover, many obesity-related SNP sites have been reported to have regional differences [13, 59], such as *IRS2*, a gene significantly associated with diabetes. It has been found by GWAS to be highly conserved in the African population, but heterogenic in Asian and European populations [24]. Furthermore, natural selection often happened in 10,000–15,000 years, when humans spread out of Africa [60]. Besides, a great deal of metabolic disorder-related genes has been reported to be an adaptation to the climate [61], implicating that climate is sufficient to pose selective pressure. The abovementioned facts all lay a foundation for the demonstration of the maladaptation viewpoint.

Sellayah’s demonstration mainly relies on the *UCP1* gene. *UCP1* is highly expressed in brown adipose tissue (BAT). When it turns cold, BAT burns stored triglycerides into glycerol and fatty acids. The latter enter into mitochondria in BAT, activate UCP1, uncouple oxidative phosphorylation with ATP synthesis, and eventually release thermal energy [62]. Of note, the abovementioned thermogenesis process involves the consumption of lipids. In the meantime, it is known that *UCP1* plays a key role in human adaptation to cold climates [63], and multiple GWAS have discovered SNP sites on *UCP1.* Variants show significantly different body fatness, especially 3826 A/G on its promotor [64–68], but related studies are all confined to a single population, lacking analysis of the regional difference of SNP sites on *UCP1* among different populations.

Similar to the thrifty gene hypothesis, the maladaptation viewpoint also needs to prove that climate is influential enough to cause the current obesity rate. Infants have a lot of BAT, which is essential for thermogenesis [69, 70], and premature infants often die from BAT’s poor development [71, 72]; therefore, Sellayah believes that climate has a higher *k* value than famine and can reach 0.1 (the author “asserted” the value without detailed demonstration), which is effective enough to pose selective pressure according to Haldane’s formula.

Sellayah compared the prediction and the fact to check the credibility of the maladaptation viewpoint. According to the maladaptation viewpoint, populations living in cold climates are expected to have a low obesity rate, as they should have been selected to increase metabolic rate to produce more heat, i.e., burn more fat, and vice versa. The fact is that in Scandinavian countries (whose ancestors adapted to extreme cold climate), the obesity rate is far lower than in other countries in Europe, even though they share similar diets [73, 74]; likewise, Caucasian and East Asian populations have far lower obesity rates than African American and Spaniards [58, 75]. Moreover, African Americans generally have a lower metabolic rate [76, 77], while Inuit people, who live near the Arctic Circle, have a higher metabolic rate [78]. Above all, the facts support the correctness of the maladaptation viewpoint.

There are mainly two kinds of criticism. First, the genes in the maladaptation viewpoint have little impact on obesity. The knockdown of *UCP1* has been reported to have a complex effect on BMI in different temperatures [79, 80], rather than a simple positive correlation. Besides, obesity-related genes found in GWAS have nothing to do with BAT [12–14, 81, 82]. Second, the causal relationship between obesity and thermogenesis is reversed. In other words, the less fat storage, the more heat dissipation, the more requirement for thermogenesis, and therefore the more reliance on BAT burning fat [17]. However, the criticism of causal relationship does not challenge the maladaptation viewpoint fundamentally, because the criticism itself is also a maladaptation to climate.

## Perspective

Despite the long debate as to how to explain the obesity epidemic from an evolutionary genetic perspective, neither theoretical nor factual evidence is exclusive. For the drifty gene hypothesis, the current model still neglects a lot of factors, such as obesity-related diseases; the five unknown parameters also reduce the value of the model. For the thrifty gene hypothesis and the maladaptation viewpoint, although both have corresponding supporting genes, *miR-128–1* and *UCP1*, more factual evidence is needed to strengthen the importance of the hypotheses.

Further studies on the thrifty gene hypothesis, the drifty gene hypothesis, and the maladaptation viewpoint will have important value for medicine. At present, obesity has become a problem that affects over six billion people, with no effective drug currently available. The demonstration of the abovementioned hypotheses will indicate that people of different genetic backgrounds fit a different diet. So instead of today’s one-size-fits-all therapy, perhaps precision medicine is a better strategy for treating obesity. To be specific, nutrition should be customized according to different genomes to deal with obesity more efficiently.

## Conclusion

A variety of ideas have been proposed as to how to explain the present obesity epidemic. To better understand the issue, a comprehensive review is needed. However, the related reviews have all been written by supporters of certain ideas and have not been updated for over five years. Therefore, this mini-review combines the typical theoretical models with the latest factual evidence from a neutral stand and overviews the thrifty gene hypothesis, the drifty gene hypothesis, and the maladaptation viewpoint from both logical and factual levels. What is worth noticing is that, although the debate is fierce, these three ideas are, in principle, not mutually exclusive. The key point of the thrifty gene hypothesis is that famines served as natural selection toward the underweight, and those who could take full use of food were more likely to survive and pass their genome. For the drifty gene hypothesis, the core is that mastering tools and fire enabled humans to eliminate the pressure from predators, and thus arbitrary gene mutation and drift could accumulate in the long human history. In summary, the thrifty gene hypothesis targets the underweight, while the drifty gene hypothesis targets the overweight. As for the maladaptation viewpoint, genes are directly associated with climate rather than with body weight. Therefore, the demonstration of any of these hypotheses does not mean the denial of the other two. Future studies need to further perfect the model and provide more factual evidence to successfully prove the hypotheses and eventually instruct humans to better understand and tackle obesity.

## Data Availability

Not applicable.

## Abbreviations

BMI: Body mass index
NCD: Non-communicable chronic disease
T2D: Type 2 diabetes
mya: Million years ago
GWAS: Genome-wide associated studies
SNP: Single nucleotide polymorphism
iHS: Integrated haplotype score
XP-EHH: Cross-population extended haplotype homozygosity
BAT: Brown adipose tissue
